# Supplementary figures and images for: Epigenetic modification mechanism of histone demethylase KDM1A in regulating cardiomyocyte apoptosis after myocardial ischemia-reperfusion injury
Source: PeerJ. 2022 Aug 5;10:e13823. doi: 10.7717/peerj.13823 (PMC9359132; doi:10.7717/peerj.13823)

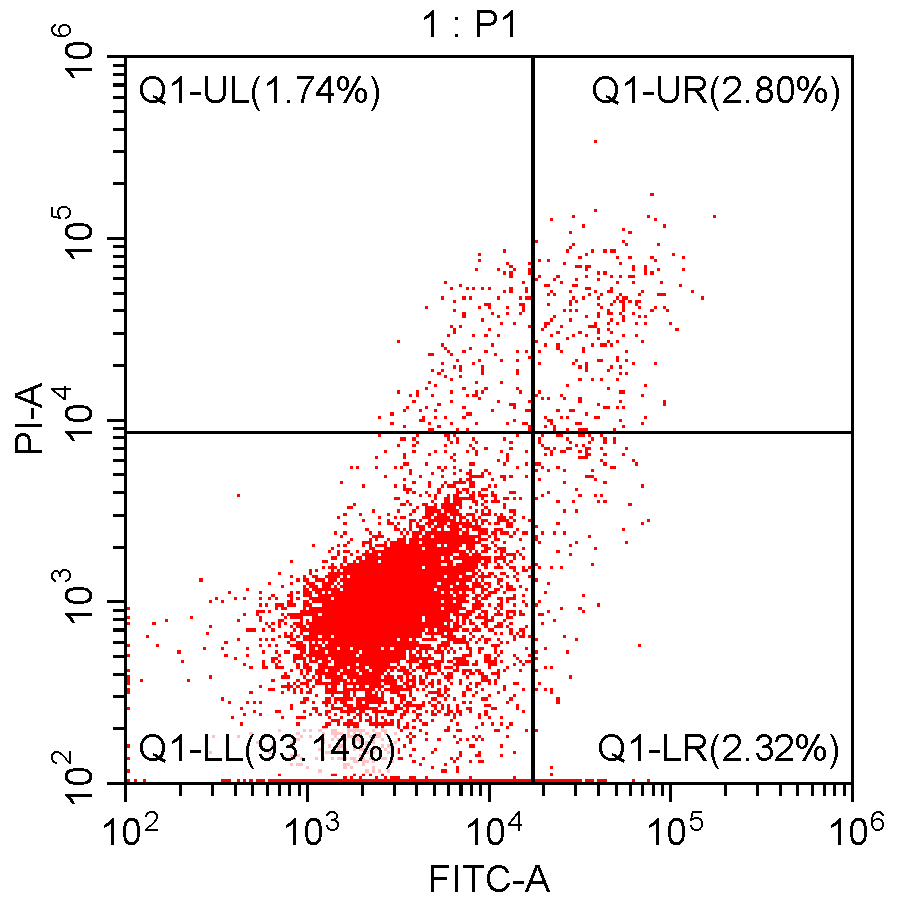

Supplement: Supplemental Information 1 [file peerj-10-13823-s001.zip › Supplemental File/Figure 1/figure of apoptosis/Blank/1_Plot1.bmp]

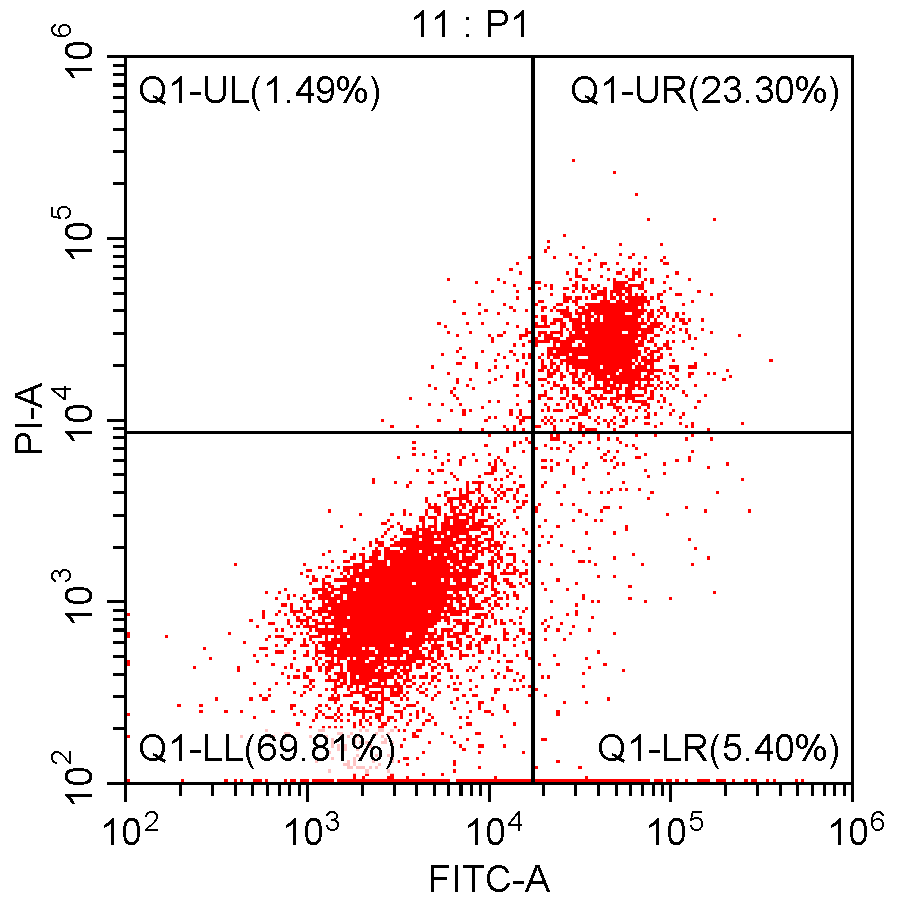

Supplement: Supplemental Information 1 [file peerj-10-13823-s001.zip › Supplemental File/Figure 1/figure of apoptosis/HR/11_Plot1.bmp]

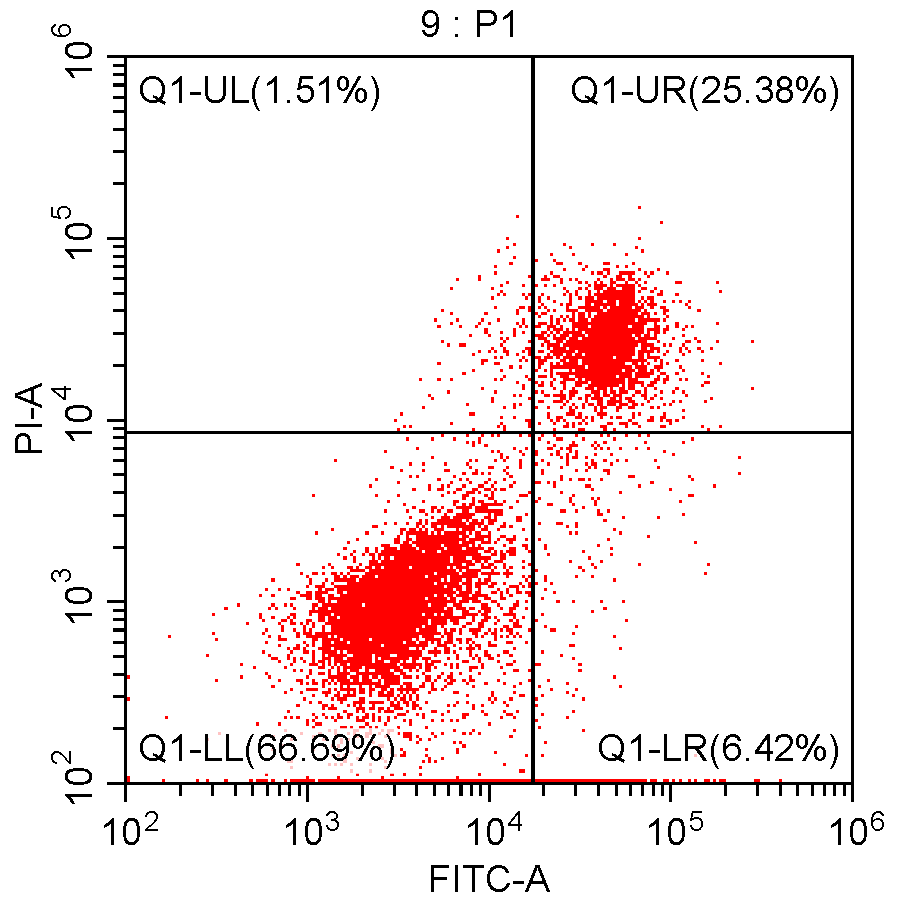

Supplement: Supplemental Information 1 [file peerj-10-13823-s001.zip › Supplemental File/Figure 2/figure of apoptosis/HR/9_Plot1.bmp]

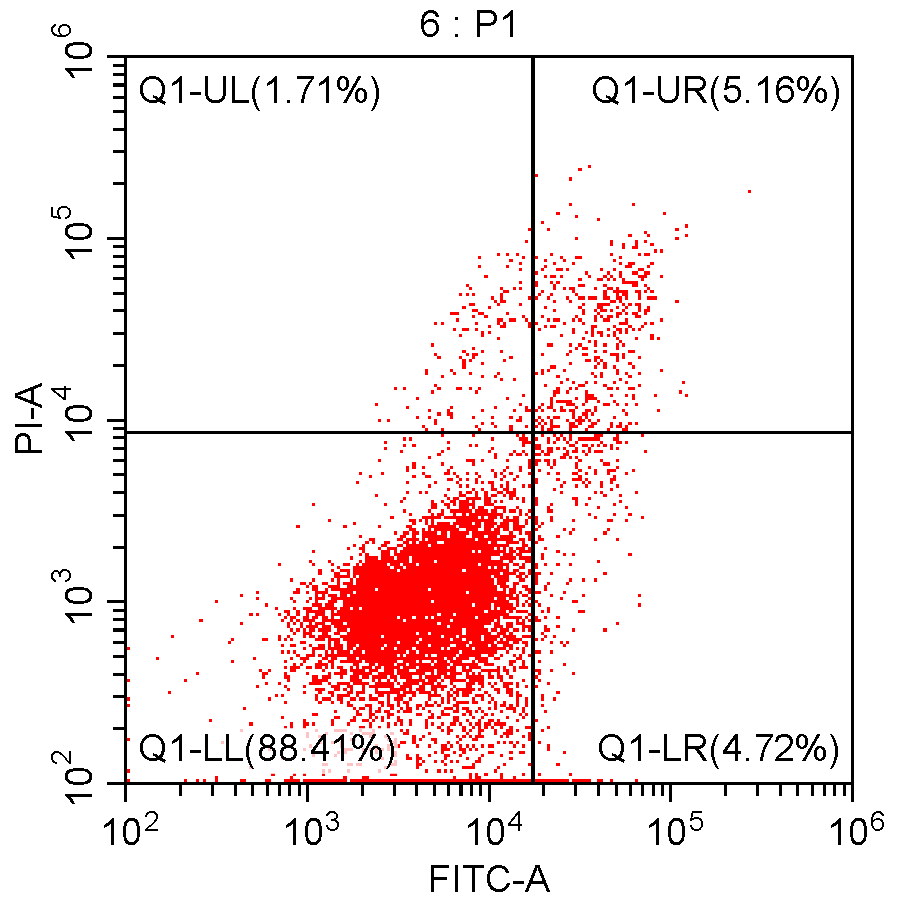

Supplement: Supplemental Information 1 [file peerj-10-13823-s001.zip › Supplemental File/Figure 2/figure of apoptosis/HR + oe-KDM1A/6_Plot1.bmp]

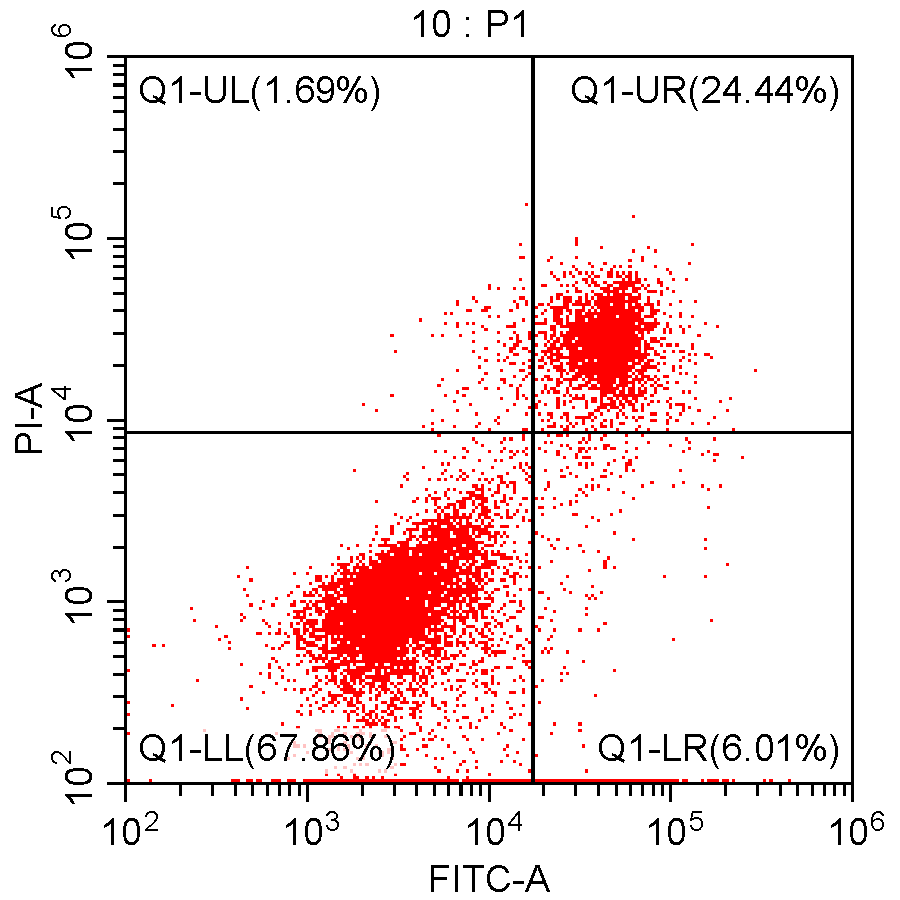

Supplement: Supplemental Information 1 [file peerj-10-13823-s001.zip › Supplemental File/Figure 2/figure of apoptosis/HR + oe-NC/10_Plot1.bmp]

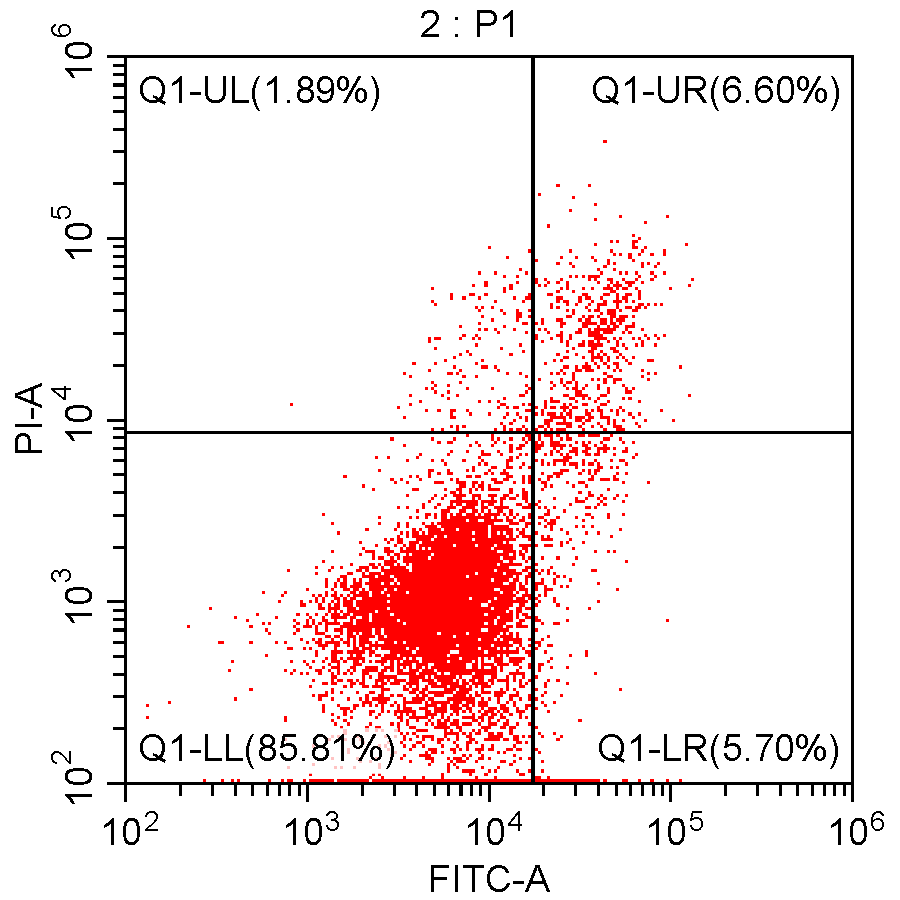

Supplement: Supplemental Information 1 [file peerj-10-13823-s001.zip › Supplemental File/Figure 4/figure of apoptosis/HR + oe-KDM1A/2_Plot1.bmp]

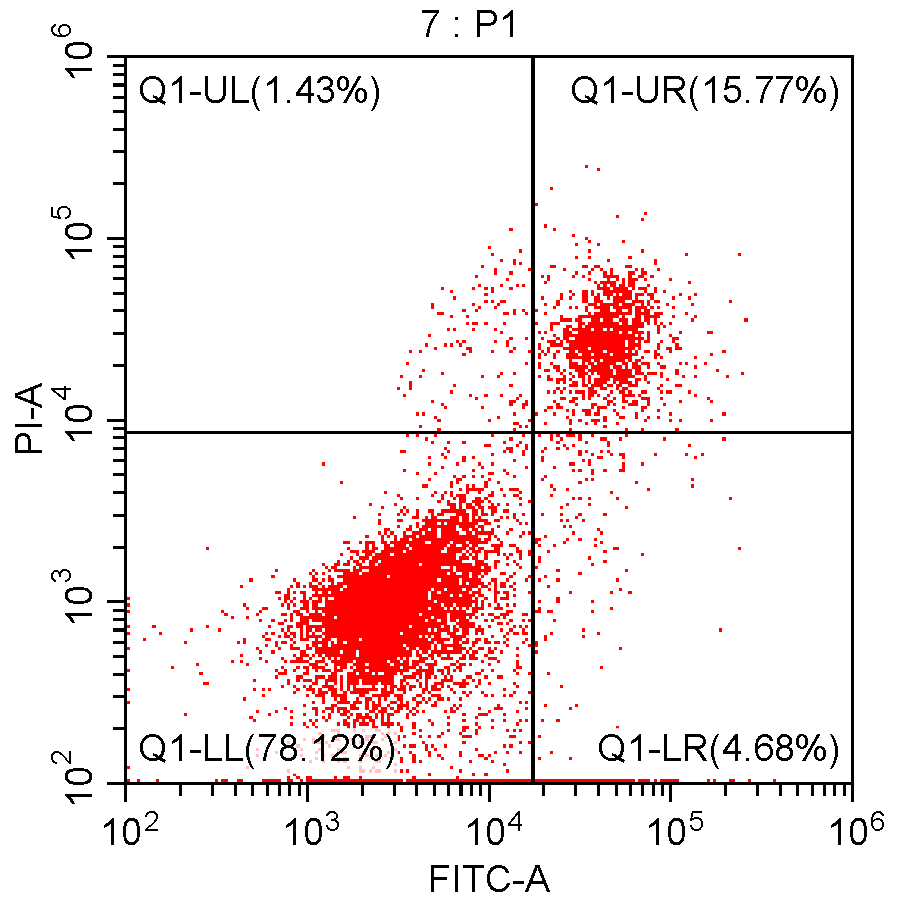

Supplement: Supplemental Information 1 [file peerj-10-13823-s001.zip › Supplemental File/Figure 4/figure of apoptosis/HR + oe-KDM1A + CPI/7_Plot1.bmp]

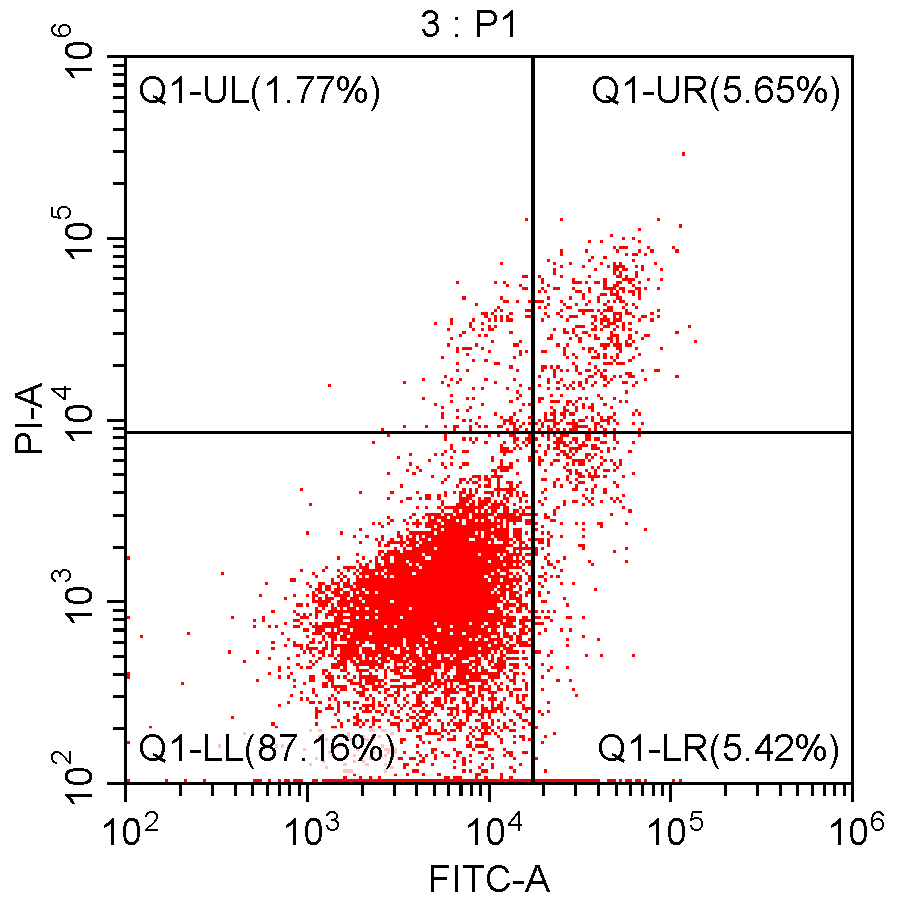

Supplement: Supplemental Information 1 [file peerj-10-13823-s001.zip › Supplemental File/Figure 4/figure of apoptosis/HR + oe-KDM1A + DMSO/3_Plot1.bmp]

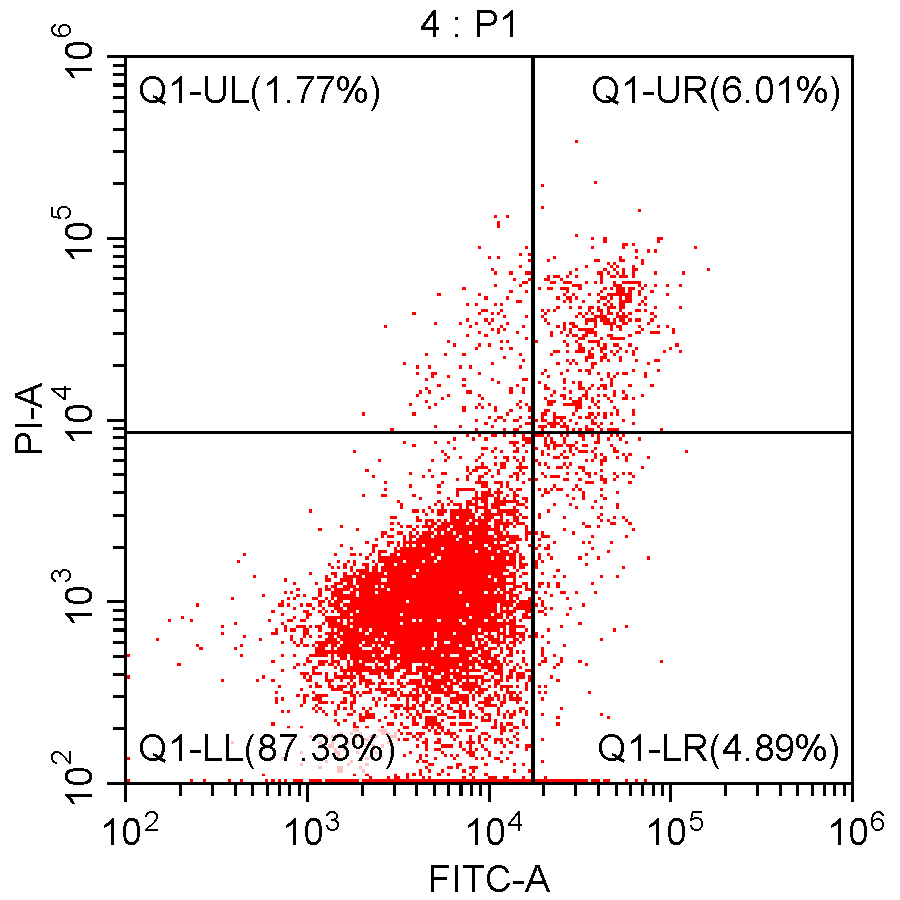

Supplement: Supplemental Information 1 [file peerj-10-13823-s001.zip › Supplemental File/Figure 5/figure of apoptosis/HR + oe-KDM1A/4_Plot1.bmp]

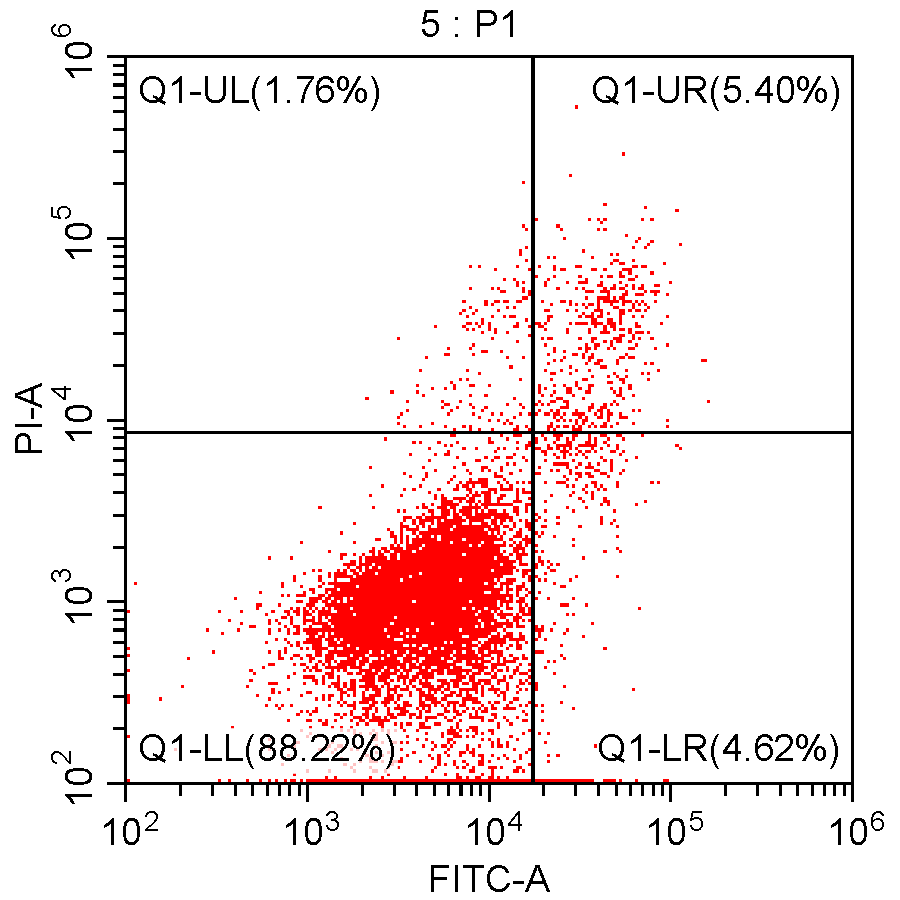

Supplement: Supplemental Information 1 [file peerj-10-13823-s001.zip › Supplemental File/Figure 5/figure of apoptosis/HR + oe-KDM1A + oe-NC/5_Plot1.bmp]

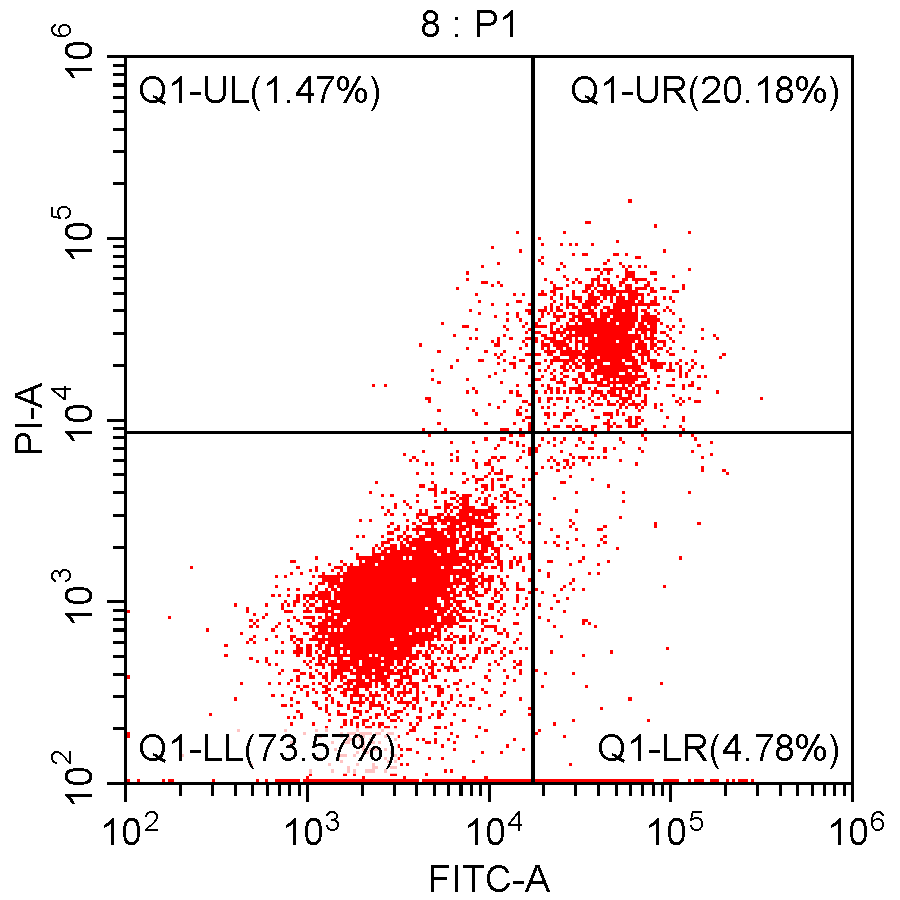

Supplement: Supplemental Information 1 [file peerj-10-13823-s001.zip › Supplemental File/Figure 5/figure of apoptosis/HR + oe-KDM1A + oe-SOX9/8_Plot1.bmp]

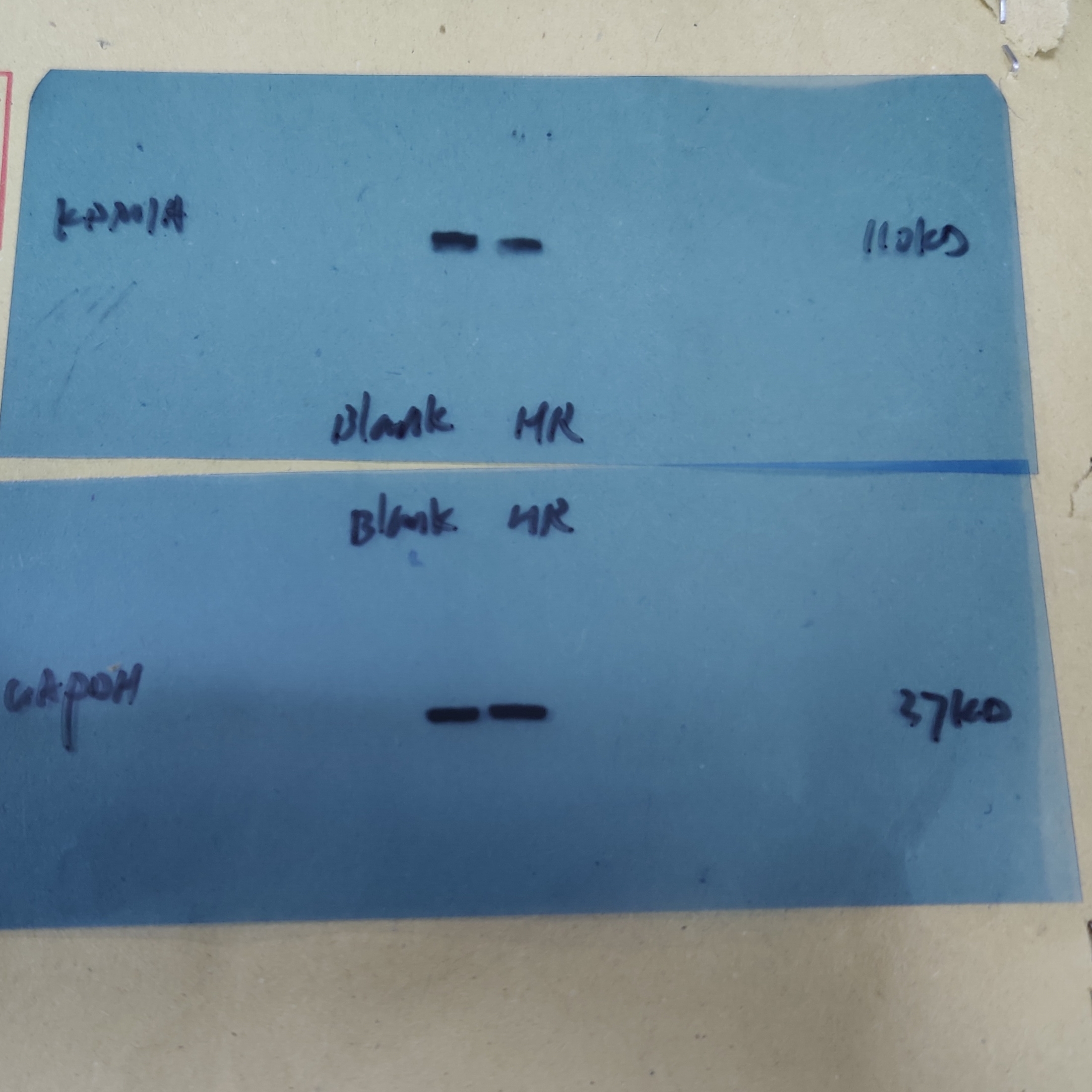

Supplement: Supplemental Information 2 [file peerj-10-13823-s002.zip › Western blot/Western blot gels/Figure 1B.jpg]

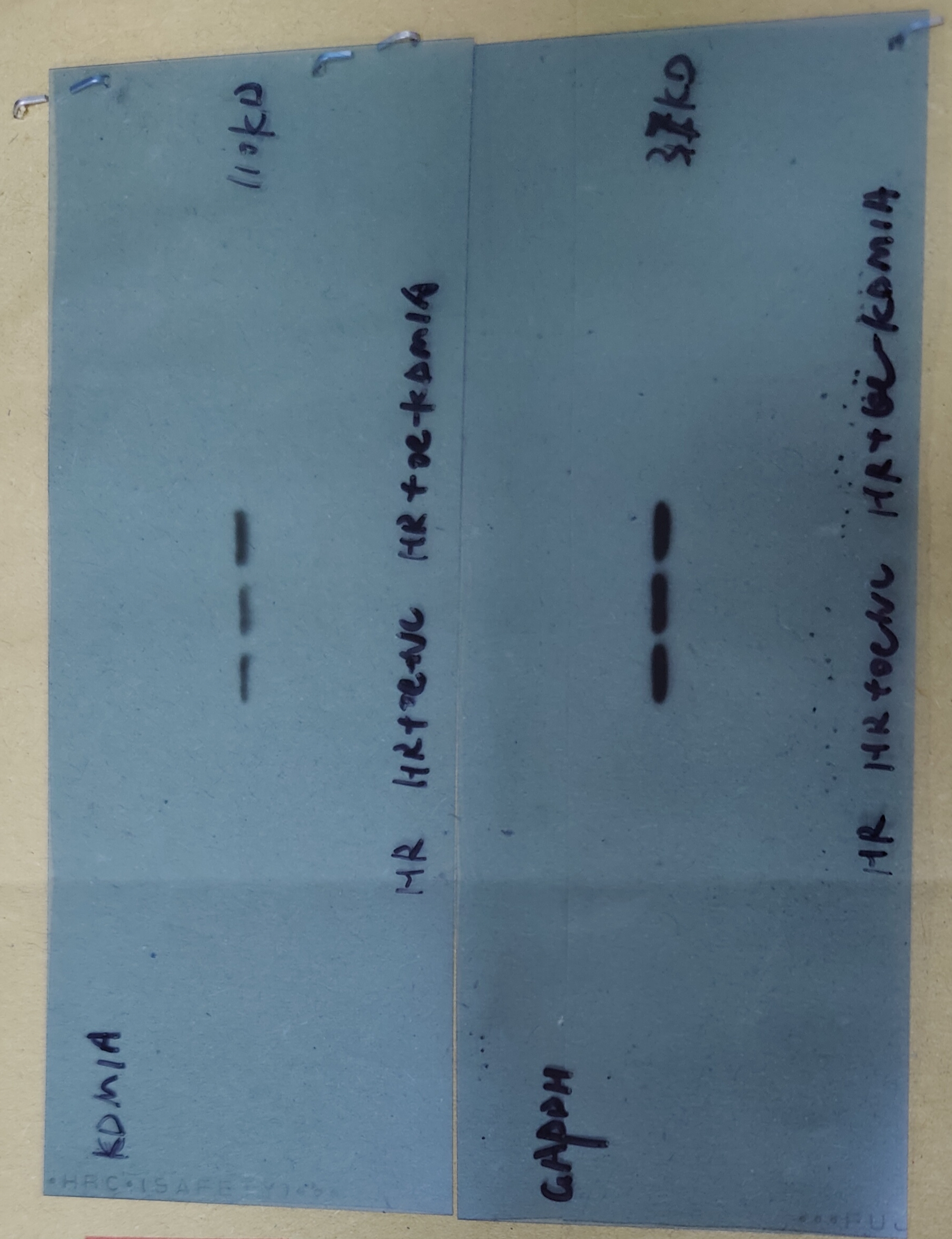

Supplement: Supplemental Information 2 [file peerj-10-13823-s002.zip › Western blot/Western blot gels/Figure 2B.jpg]

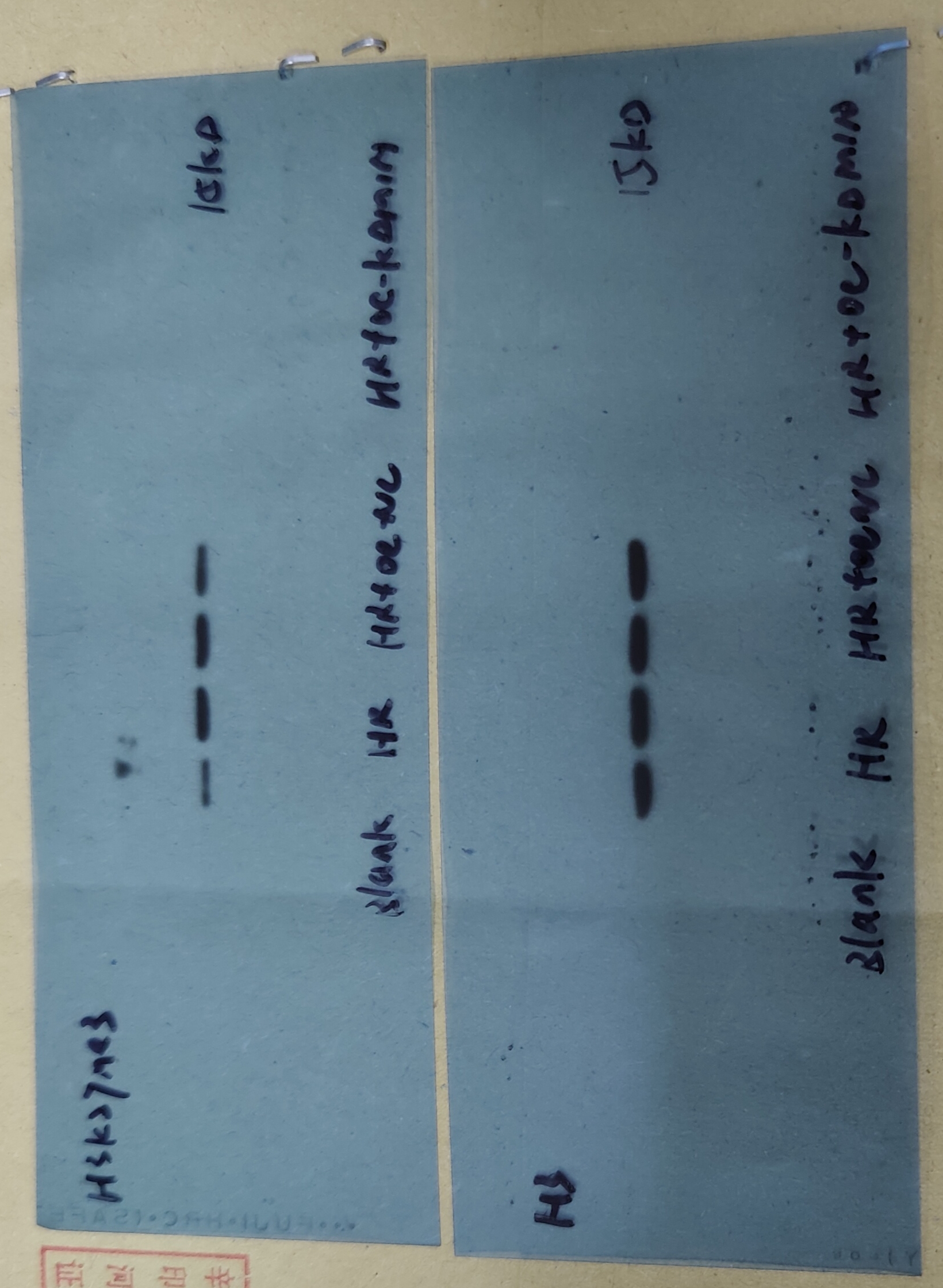

Supplement: Supplemental Information 2 [file peerj-10-13823-s002.zip › Western blot/Western blot gels/Figure 3A.jpg]

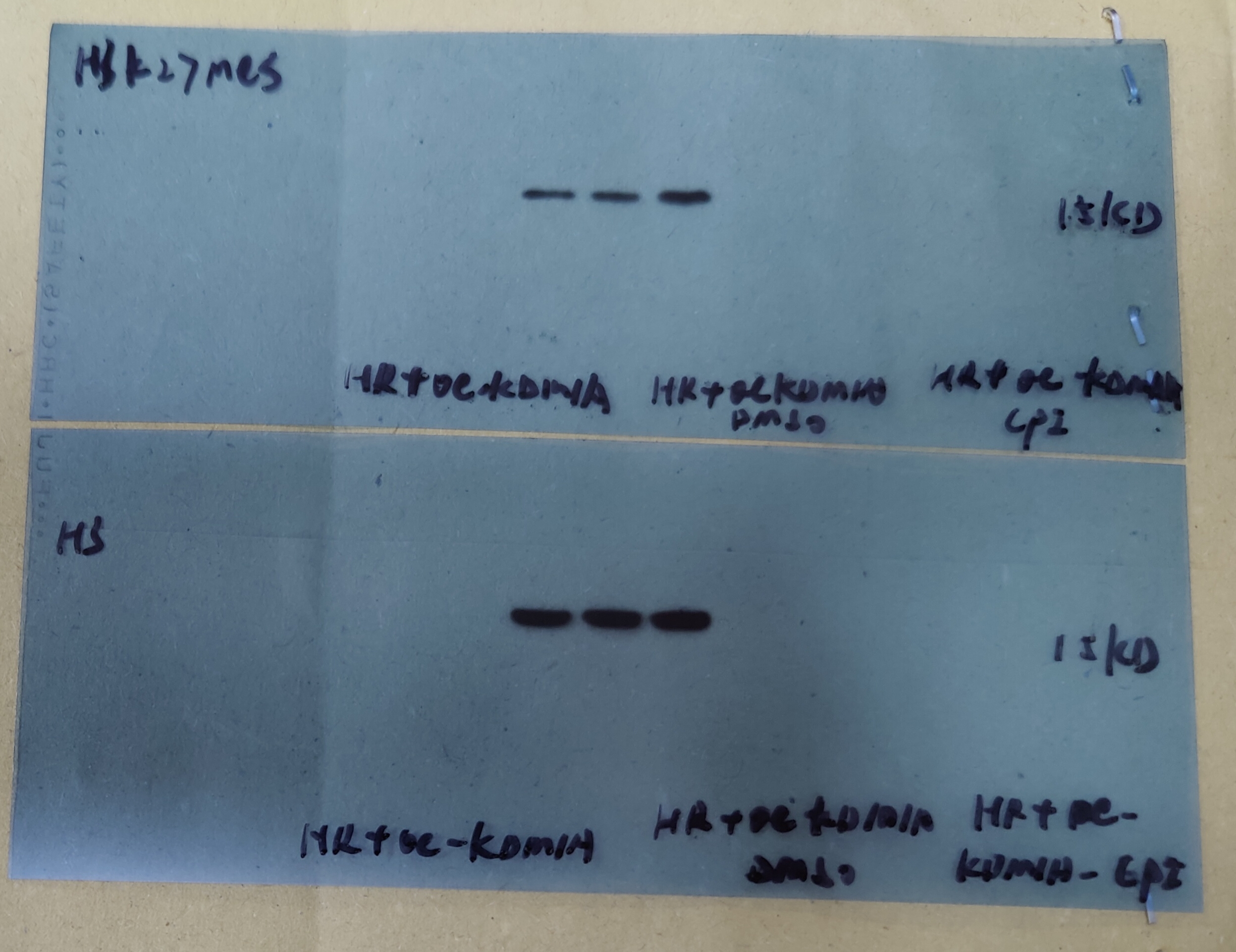

Supplement: Supplemental Information 2 [file peerj-10-13823-s002.zip › Western blot/Western blot gels/Figure 4A.jpg]
